# Comparison of the Safety and Efficacy of Laser Versus Pneumatic Intracorporeal Lithotripsy for Treatment of Bladder Stones in Children

**DOI:** 10.3390/jcm11030513

**Published:** 2022-01-20

**Authors:** Ziad H. Abd, Samir A. Muter

**Affiliations:** 1 Department of Surgery, College of Medicine, University of Anbar, Ramadi 31001, Iraq; 2 Department of Surgery, College of Medicine, University of Baghdad, Baghdad 10001, Iraq; Samir.muter@yahoo.com

**Keywords:** laser lithotripsy, pediatric urolithiasis, pneumatic lithotripsy, vesical stone

## Abstract

This study aimed to compare the safety and efficacy of laser lithotripsy and pneumatic lithotripsy, the two most commonly used transurethral lithotripsy methods for treating bladder stones in children in Iraq. Between January 2013 and December 2016, 64 children with bladder stones were included in this prospective randomized study, after ethical committee approval and written consent from the children’s parents or caregivers were obtained. Patients were assigned randomly by computer software to two groups treated with either pneumatic cystolithotripsy or laser lithotripsy. A 9 Fr. semirigid ureteroscope was used to pass the lithotripter through and fragment the stone. A catheter of 8–12 Fr. was then introduced and kept in place for 24 h. All children were hospitalized for 24 h, and the catheter was removed the next morning. Outpatient follow-up was maintained for 6–12 months. In terms of operation outcomes and complications, the laser lithotripsy group had a significantly longer duration of operation (74.5 ± 26.6 min vs. 51.5 ± 17.2 min, *p* = 0.001), whereas the number of patients requiring an extended hospital stay was significantly higher in the pneumatic lithotripsy group (48.5% vs. 16.1%, *p* = 0.006). Moreover, pneumatic lithotripsy was associated with a significantly greater risk of having at least one adverse effect (64% greater than that in the laser group). Stone clearance rates did not significantly differ between treatment groups. In conclusion, both pneumatic and laser lithotripters can be used to treat children with bladder stones with high efficacy and safety.

## 1. Introduction

Endemic bladder stones in children account for a substantial urologic workload in hospitals in many developing countries despite having nearly disappeared in developed countries as early as 1920 [1]. Iraq and the entire Middle East lies in the Afro-Asian “stone belt”, a region with an increased incidence of bladder stones [2]. Compared with those in adults, bladder stones in children are associated with metabolic, nutritional and genetic factors. Dehydration and infection play major roles in bladder stone formation in children [3,4]. Ammonium urate and calcium oxalate stones have been attributed to poor nutrition and low-protein and high-carbohydrate diets [5]. Infants in underdeveloped countries are mainly fed human breast milk and cereals, particularly polished rice, both of which contain low phosphorus content. Low phosphorus diets increase urinary ammonium content [5], thus potentially explaining the high prevalence of bladder stones in developing countries.

Historically, bladder stones were treated with open cystolithotomy, although this procedure is currently rarely needed. Percutaneous cystolithotomy and extracorporeal shock-wave lithotripsy were introduced later during the last century [6]. Although cystolitholapaxy and cystolithotripsy (CL) are the standard treatment modalities for adult bladder stones, a transurethral approach to bladder stones in children was previously not recommended because of a lack of availability of small instruments and the risk of injuring the narrow urethra and causing lifelong urethral stricture disease [7]. Shock-wave treatment of bladder stones in children has been associated with a high retreatment rate and difficulties in passing the stone fragments [8]. However, after smaller pediatric cystoscopes and pneumatic and laser lithotripters became available, a rapid shift toward the use of less invasive transurethral CL for treating pediatric bladder stones resulted. In 1994, Shokier reported the safe use of a pneumatic lithotripter to fragment bladder stones in children [9]. Subsequently, in 2005, Ramakrishnan et al. used a laser (Ho:YAG) for the same purpose and reported the method to be safe and successful [10]. Since then, many studies have confirmed the safe and efficient use of both modalities in the treatment of pediatric bladder stones. Recently, minimally invasive surgery (MIS) has shown to be safe and effective in removing big bladder stones [11]. The superior advantage of MIS is the possibility of removing bladder stones through the navel, utilizing an endobag, without crushing them [11]. Moreover, robot-assisted laparoscopic surgery has demonstrated efficacy in the treatment of large-volume stones, especially in conditions requiring simultaneous reconstruction [12]. However, robotic surgery in pediatric bladder stones or urolithiasis still needs more evidence to be implemented within the practice [13,14].

Although pneumatic lithotripters have been available in Iraq for many years, laser lithotripters have only recently been introduced and begun to be used by a few urologists. However, other urologists were hesitant to use the laser with concerns related to costs and questionable superiority over pneumatic lithotripters.

We hypothesized that laser CL has equal efficacy and safety compared to pneumatic CL in pediatric bladder stones and that the higher cost of the procedure is outweighed by a lower complication rate and shorter hospital stay.

This study aimed to compare the safety and efficacy of the two transurethral lithotripters most commonly used in the treatment of pediatric bladder stones in Iraq in terms of efficacy and safety. This study has a superior contribution to the field given its prospective design and large sample size compared to previous trials.

## 2. Materials and Methods

Between January 2013 and December 2016, all children aged 12 years or less and diagnosed with single bladder stones were enrolled in this prospective randomized study after obtaining the ethical committee approval from University of Anbar, College of Medicine with an ethical code of 128EA and written consents from the children’s parents or caregivers. All patients belonged to a single ethnic group, Arab.

Exclusion criteria were the presence of active urinary tract infection, multiple bladder stones, urinary tract functional or anatomical abnormality, or previous urinary tract surgical intervention.

After taking a full medical history and performing full clinical examinations on all children, we sent them for laboratory investigations. Laboratory investigations included full blood count, renal function test (electrolytes, urea and creatinine) and urinalysis. Urine culture was ordered only if the urine test showed WBCs > 3 or a UTI was clinically suspected because of the limited availability of culture media in the hospital at the time the study was conducted. Patient imaging was used to make the initial diagnosis and then reviewed by the treating consultant, and additional imaging was arranged. All patients were imaged using US and X-ray KUBs. A contrast study of the lower or upper urinary tract to exclude functional or anatomical abnormalities was arranged when clinically indicated.

All relevant data were recorded on SPSS-21 for analysis. The stone size was entered as the largest dimension measured by the US and X-ray KUB. When there was a discrepancy in the measurement between the two methods, the largest diameter measured in either of them was used.

Computer software was used to randomize patients into two treatment groups. In the first group, patients were treated with pneumatic CL, whereas patients in the second were treated using laser CL. The patient’s assignment into one of the treatment groups was recorded in the patient’s medical record and disclosed to the treating surgeon on the morning of the operation.

All patients were fasted for 6–8 h and admitted to the hospital on the day of surgery. According to the hospital rules, all pediatric patients were treated at the start of the list and according to their age.

All procedures were conducted by one senior urologist utilizing general anesthesia while patients were in a semilithotomy position. A single prophylactic dose of intravenous antibiotic (cephazolin 30 mg/kg, unless urine culture and sensitivity or patient allergy profile dictate another antibiotic) was administered at time of anesthesia induction.

Initially, urethrocystoscopy was performed with an 8 Fr. Pediatric cystoscope to exclude urethral/bladder abnormalities and document the presence of the stones. A 9 Fr. semirigid ureteroscope was then used to pass the lithotripter through and fragment the stone (0.8 mm tip cystolithoclast, Karl Storz, or 365 µm quartz fiber Ho:YAG, CALCULASE II, Karl Storz). No energy was applied until the probe was clearly observed to be in contact with the stone. Pneumatic energy was applied in a pulsatile manner while laser was applied with 6–10 Hz, 1 J (for fragmentation), and 10–15 Hz, 0.5–0.8 J (for dusting). Normal saline was used for irrigation, and large stone fragments were extracted with a Storz grasper.

Stone fragmentation continued until stone-free status was achieved or it was not safe to continue because of severe hematuria impairing vision. Stone-free status was confirmed intraoperatively by comparing the stone fragments with the probe tip and postoperatively using US/X-ray KUB one week after the operation. All fragments larger than twice the probe tip were either fragmented more or removed with a stone grasper.

Operation time was calculated from the insertion of the ureteroscope for the first time to the removal of the last cystoscope/ureteroscope after stone fragments retrieval and/or washout.

An 8–12 Fr indwelling urethral catheter (IDC) was introduced at the end of the procedure and later patients were admitted into the ward and kept on oral paracetamol every 6 hours, with instructions for a trial of void (TOV) at 7 AM in the next morning. Patients were allowed to restart their oral intake once fully recovered from anesthesia, and no patient received IV fluid postoperatively. Outpatient follow-up continued for at least 6 months after discharge from the hospital. Children who failed the initial TOV were recatheterized and had their bladders examined by US to exclude presence of significant blood clots and then given a second TOV after 24 h. Any temperature greater than 37.5 °C was considered significant, and those patients were started on oral antibiotics after sending urine samples for culture and sensitivity. The degree of macroscopic hematuria was estimated visually; when it was more than a faint rose in color, it was considered significant and an indication to postpone patient discharge from hospital. Data were tabulated and analyzed statistically in SPSS-21 (Chicago, IL, USA). Qualitative data were expressed as percentages, and quantitative data were expressed as mean ± standard deviation (SD).

The primary outcome of the study was to compare the stone-free rates in the treatment group. The complication rate in both groups was compared also and regarded as a secondary outcome.

## 3. Results

A total of 73 children were diagnosed with bladder stones during the study period, but only 64 were enrolled in the study. Nine patients were excluded, five of them due to presence of urinary tract infection, three due to multiple stones and one due to neuropathic bladder.

Of the 64 children who completed the study, 33 were treated with pneumatic CL and 31 were treated with laser CL. Demographic and baseline parameters in both groups were comparable, as evident in Table 1. In regard to the diet status of the patients, all patients followed a Middle Eastern Iraqi diet, of which the main components are wheat, barley and rice, dairy products and red meat (lamb and beef).

Regarding the operation outcomes and complications, the laser lithotripsy group had a significantly longer duration of operation (74.5 ± 26.6 min vs. 51.5 ± 17.2 min, *p* = 0.001), whereas a significantly greater proportion of patients required an extended hospital stay in the pneumatic lithotripsy group (48.5% vs. 16.1%, *p* = 0.006). Moreover, pneumatic lithotripsy was associated with a significantly greater risk of having at least one adverse effect (64% greater than that in the laser group). Of the 31 patients who were treated with laser CL, only five (16.12%) required more than one day of hospitalization and each of those five required only 2 days. On the other hand, for those who were treated with pneumatic CL, 16 (48.48%) required more than one day of hospitalization, with an average stay of 2.64 days (eight patients for 2 days, five for 3 days and three for 4 days).

No significant difference was found between the two treatment groups in relation to stone clearance rates and all other studied adverse outcomes, as described in Table 1. Hematuria was found in four patients in each group (12%), and none of the patients required transfusion. No patient from those who had significant hematuria that delayed IDC removal and TOV was found to have a significant intravesical blood clot that required washout or a further delay of TOV. 

Two patients in the pneumatic CL group and one in the laser CL group had significant residual stones and required a second procedure to clear the stone. All three patients had severe hematuria by the end of the procedure, which significantly impaired visualization.

In long-term follow-up, no patients in either group developed symptoms or signs suggestive of urethral stricture that required further investigation. Moreover, two patients re-presented with recurrent bladder stones in the pneumatic CL group and one did in the laser CL group.

A Mann–Whitney U test was used to analyze the scores of adverse outcomes in the two groups, and the difference was found not to be significant. In addition, a larger stone size was found to be associated with having at least one adverse outcome in both groups (Table 2).

A multiple regression model was created and found to predict the risk of developing at least one adverse outcome with an accuracy of 84.4%. According to this model, pneumatic lithotripsy was associated with a significant increase in the risk of developing at least one adverse postoperative outcome (5.7 times that of the laser lithotripsy group) after adjustment for the possible confounding effects of age and stone size (Table 3). Age was not significantly associated with the risk of developing adverse outcomes (Table 3).

## 4. Discussion

Transurethral cystolitholapaxy and CL have been considered the first-line treatments for bladder stones in adults for decades; however, the use of these techniques in children was delayed for many years, during which open CL and percutaneous CL were the first-line treatment choices. These two surgical techniques are associated with significant morbidity, including scar formation, prolonged catheterization and longer hospital stays. Bowel injury is a more serious complication specific to percutaneous CL.

Later, the availability of pediatric cystoscopes and ureteroscopes, as well as highly effective intracorporeal lithotripsy devices with miniprobes, enabled the feasibility of transurethral CL in children [3,9,10,15]. Currently, pneumatic, laser and, to a lesser extent, electrohydraulic lithotripters are used to fragment stones in children [16]. Recent advances in robotic surgery will open the doors for even less invasive robotic-assisted removal of bladder stones in pediatric patients [12,13,14].

The safety and efficacy of pneumatic lithotripters in treating bladder stones in children were tested and reported by many authors following their initial use by Shokier [3,9,14,17]. Ho:YAG lasers enabled a major breakthrough in the management of stone disease in general. These lasers are safe and effective and can fragment many types of stones [10,17].

Each of these two lithotripter types has pros and cons. Pneumatic lithotripter probes are relatively inexpensive, and the energy generators are easy to maintain. The main problem associated with the use of pneumatic lithotripters is stone migration, owing to the ballistic mechanism used to fragment stones. Laser lithotripters, in contrast, are much more expensive. The probes are usually disposable, and the laser generators are costly to maintain. Laser, however, has the advantages of being able to fragment stones into submillimeter pieces without significant stone migration and of eliminating the need to use graspers to extract larger stone fragments [18].

This prospective study aimed to compare the safety and efficacy of pneumatic and laser lithotripters in treating children with bladder stones. The patient age, sex and stone size were comparable between the two treatment groups and were not expected to confound the analysis.

Because operation time is an important factor in any surgical procedure, we compared the time required to fragment stones with pneumatic and laser CL. Laser stone fragmentation required significantly longer durations (74.5 vs. 51.5 min). Gangkak and coworkers reported no significant difference in operation time between laser and pneumatic lithotripsy (36.6 vs. 35.5) [17]. They also reported a much shorter operation time than that in our study. This discrepancy might be explained by our limited experience in using laser lithotripsy, given that this method was newly introduced to our center, as well as by our practice of reusing the laser fibers to conserve our limited resources, thus potentially decreasing the laser fiber efficiency.

In comparing the outcomes of surgery, we found that the two groups had comparable stone-free rates and postoperative complications. A Mann–Whitney U test used to count the scores of adverse outcomes did not indicate any significant differences between the two groups. The only significant difference observed was that patients treated with pneumatic lithotripsy required longer hospital stays than those treated with laser lithotripsy. Only five patients (16.1%) treated with laser lithotripsy required an extended hospital stay, and all of them required one additional day. In the pneumatic lithotripsy group, in contrast, almost half the patients required an extension of their hospital stay (16 patients, 48.48%). The average hospital stays for patients who required an extended stay in the laser CL group was 2 days, compared to 2.68 days in the pneumatic CL group.

Of the 16 patients who required extended hospital stays in the pneumatic CL group, eight patients had 2 days of admission, five patients had 3 days and three patients had 4 days. Reasons for the longer-than-planned hospital stay were hematuria in four patients, failed TOV in two patients, infection in four patients and pain or parents’ anxiety in six patients. Apart from parents’ anxiety, all other causes of extended hospital stay were attributed to the surgery itself. Postoperative pain score was difficult to appreciate or measure in children, and this was the reason behind excluding pain as a postoperative complication to measure and compare in both treatment groups.

In a comparison of overall outcomes, the risk of having at least one adverse outcome was higher in the pneumatic lithotripsy group (*p* = 0.046).

The mean stone size in patients with at least one adverse outcome was significantly higher in both treatment groups and can be considered an independent predictor of adverse outcomes. Aboulela and colleagues divided patients into two groups according to stone size and treated them with laser lithotripsy and found that the operation time and complication rate significantly differed between groups [19]. A similar finding was reported by Abdul Rasheed and coworkers in patients treated with pneumatic lithotripsy [8]. The authors, however, reported a greater overall rate of complications, which may have been associated with the urethral dilation performed at the start of the procedure in that study.

The rate of urinary tract infection after pneumatic lithotripsy was four times higher than that after laser lithotripsy (4 (12.1%) vs. 1 (3.2%)), but this difference was not statistically significant (*p* = 0.36). This result may be associated with previous recurrent use of stone graspers to clear out larger stone fragments in the patients treated with pneumatic lithotripsy.

To identify factors that independently predicted the outcome, we created a multiple regression model and found that pneumatic lithotripsy and stone size were the only two factors that independently predicted the outcome. Compared with laser lithotripsy, pneumatic lithotripsy increased the risk of at least one adverse outcome by 5.7 times. The model also showed that every 1 mm increase in stone size was associated with a 75% increase in the chance of an adverse outcome. Patient age and sex of the patient were not found to be independent factors affecting the surgery outcomes.

No long-term complications were found in any of the treated patients in terms of urethral stricture or chronic lower urinary tract symptoms. Similar results were reported by most previous studies [9,10,15,17,19]. Abdul Rasheed and colleagues also reported a 3.5% rate of urethral stricture after pneumatic lithotripsy, which again might have been associated with the urethral dilation that was performed. Al-Marhoon and associates compared G and pneumatic lithotripsy to open cystolithotomy. One of their patients treated with pneumatic lithotripsy had a urethral rupture with extravasation and later developed urethral stricture [20]. In their conclusion, the authors recommend the use of laser fibers through a ureteroscope to reduce the risk of urethral injury. Javanmard and colleagues compared transurethral laser lithotriopsy with open and percutaneous cystolithotomy in treating bladder stones in children and described laser as a safe, effective and minimally invasive treatment option [21].

One of the study limitations is stone composition, which is another factor that must be considered. Some stones are difficult to fragment and take too long to be destroyed. Unfortunately, our study, like most of the studies related to this topic, does not report on the stone composition. In a trial to overcome this issue, we performed an X-ray KUB to check for stone radiopacity. However, this, unfortunately, was not very helpful, as only four stones in both groups were radiopaque.

In our center, the laser machine was introduced only recently, and we, therefore, face technical and financial hurdles in maintaining the energy generator and the laser fibers. Because our public hospital has a high workload and limited resources, we must consider these hurdles when choosing treatment modalities, despite the apparent superiority of laser over pneumatic lithotripsy.

## 5. Conclusions

Pneumatic and laser lithotripsy are equal with regard to stone clearance rate. However, pneumatic lithotripsy has a shorter operating time, longer hospital stays and a more adverse effect. Laser lithotripsy appears to be relatively superior to pneumatic lithotripsy in terms of being associated with fewer complications, but its cost might limit its use. In choosing the treatment modality, stone size is the most important factor to consider in cases in which complications are expected.

## Figures and Tables

**Table 1:** Demographics and clinical data in both treatment groups.

Postoperative Outcomes	Pneumatic Lithotripsy (*n* = 33)	Laser Lithotripsy (*n* = 31)	*p*-Value
Age (years) (mean ± SD)	4.2 ± 2.2	3.9 ± 2.1	0.62
Sex (M:F)	30:3	30:1	0.61
Stone size (mm) (mean ± SD)	15.9 ± 4.6	15.7 ± 5.2	0.87
Duration of operation in minutes (mean ± SD)	51.5 ± 17.2	74.5 ± 26.6	**0.001**
Residual stones	2 (6.1)	1 (3.2)	1.0
Severe hematuria	4 (12.1)	4 (12.9)	1.0
Urinary retention after removal of catheter	2 (6.1)	1 (3.2)	1.0
Recurrence	2 (6.1)	1 (3.2)	1.0
Postoperative infection	4 (12.1)	1 (3.2)	0.36
Extended hospitalization	16 (48.5)	5 (16.1)	**0.006**
More than one day of IDC	10 (30.0)	6 (19.3)	0.19
Requirement for more than one session	2 (6.1)	1 (3.2)	1.0
At least one positive adverse outcome	21 (63.6)	12 (38.7)	**0.046**

Values are shown as number of patients, with percentage in parentheses, unless otherwise indicated.

**Table 2:** Differences in mean stone size between patients with at least one adverse outcome and those without adverse outcomes in both groups.

	Pneumatic Lithotripsy		Laser Lithotripsy	
	At Least One Adverse Outcome		At Least One Adverse Outcome	
	Negative	Positive	Negative	Positive
Mean stone size (mm)	13.1	17.5	12.4	21.0
Stone size range (mm)	(10–18)	(11–31)	(10–17)	(15–30)
SD	2.4	4.8	2.2	4.0
SE	0.70	1.04	0.51	1.15
*n*	12	21	19	12
*p* (*t*-test, negative vs. positive)	0.005		<0.001	

**Table 3:** Multiple regression model of the risk of developing at least one adverse outcome as the dependent variable.

	Partial OR	95% Confidence Interval OR	*p*-Value
Pneumatic lithotripsy compared with laser lithotripsy	5.7	(1.17–28)	0.031
Age	1.14	(0.81–1.59)	0.46 [NS]
Stone size (mm)	1.75	(1.33–2.29)	<0.001
Constant	0.000	(0–0)	<0.001

NS: nonsignificant; OR: odds ratio. Overall predictive accuracy = 84.4%. *p* (model) ≤ 0.001.

## Data Availability

Data can be obtained from the corresponding author based on a reasonable request.
